# Near-fatal Periprosthetic Infection with *Streptobacillus moniliformis*: Case and Review

**DOI:** 10.7150/jbji.40635

**Published:** 2020-02-21

**Authors:** Matthew Smallbones, Mohammed Monem, Marina Baganeanu, Michael Okocha, Rajesh Sofat

**Affiliations:** 1 Department of Trauma & Orthopaedics, Lister Hospital, East and North Herts NHS Trust, Lister Hospital, Stevenage, Hertfordshire, SG1 4AB, United Kingdom; 2 Department of Trauma & Orthopaedics, Brunel Building, Southmead Hospital, Southmead Road, Bristol, BS10 5NB, United Kingdom.

**Keywords:** *Streptobacillus*, * moniliformis*, periprosthetic, joint, infection, rodent

## Abstract

Case presentation of a 66 year old female with penicillin hypersensitivity, who suffered late acute periprosthetic infection of her total knee replacement. After emergency surgery and admission to intensive care, the responsible organism was later identified as *Streptobacillus moniliformis*.

This serves as the first documented case of *Streptobacillus moniliformis* prosthetic joint infection. As standard culture mediums provide an exceedingly low detection rate, 16S PCR should instead be used as the first line method of identification. As a result, its detection is largely dependent on clinicians recognising relevant factors within the patient's history, namely close contact with rodents. In a patient with penicillin hypersensitivity, carbapenems have demonstrated potential as an effective treatment strategy.

## Background

In the orthopaedic setting, prosthetic joint infection is unfortunately a common occurrence, complicating 1-2% of all joint replacements 1. Treating these infections effectively is predicated on isolating the causative organism to determine the most appropriate antibiotic strategy. Sterile joint aspiration provides 45-75% sensitivity, while intraoperative tissue samples detect an organism in 65-94% of cases. There remains a group of culture-negative prosthetic joint infections where using targeted therapy proves more challenging 2.

This article describes a case of periprosthetic infection and subsequent septic shock caused by infection of *Streptobacillus moniliformis*; a gram negative organism with an exceptionally low detection rate in standard culture mediums. To date, there are no previous cases implicating S. moniliformis in the setting of prosthetic joint infection. In current literature, penicillins are the first line treatment, but the following case demonstrates successful use of carbapenems to eradicate infection in a penicillin- sensitive individual.

## Case

The patient was a 66-year-old female, with a known type-1 hypersensitivity to penicillin-based antibiotics. She had a right unicondylar knee replacement performed in 2011, which was later revised to a total knee replacement in 2014 due to progressive osteoarthritis. Both procedures were uncomplicated, with no concerns of note during her recovery and rehabilitation.

She lived independently with three cats, and worked full-time in a mail distribution centre. Despite a brief period of opioid misuse as a young adult, she remained a non-smoker with minimal alcohol intake for most of her adult life. She had no previous blood-borne or immunosuppressive viruses, and no further relevant medical history.

On Day 0, the patient was bitten on her right thumb by a small rodent, earlier brought into the home by her cat. Although two skin puncture marks were visible, she did not feel there was any acute cause for concern - and dressed the wound using a simple plaster.

Eight days later, she presented to the Emergency Department with progressive inflammation of her thumb. She also reported acute-onset right knee inflammation, which had appeared unprovoked over the past 48-72 hours. Unfortunately, she self-discharged before a full assessment could be performed.

Approximately 48-hours later, she returned to hospital with progressive deterioration of her right knee - now unable to weightbear through the intensity of her pain. She was fully orientated (AMTS 10/10), but appeared systemically unwell; experiencing pyrexia, sweating and intermittent rigors.

Her thumb was now erythematous and tensely swollen, with development of a soft tissue abscess. There was no indication of local joint involvement or proximal tracking. Her right prosthetic knee was hot, with a moderate effusion and severe generalised tenderness. Range of movement was restricted from 30˚ to 80˚ passive flexion, limited by pain. She reported no other joint problems.

Her blood tests were immediately concerning for infection, with a white cell count 19.2 x10^9^/L and C-reactive protein 353 mg/L. In addition, she had a significant acute kidney injury with creatinine 158 μmol/L and urea 19.9 mmol/L. Plain radiographs of the right thumb demonstrated soft tissue swelling, but no bony abnormality. X-rays of the right knee showed significant soft tissue effusion and visible osteolysis to suggest loosening of the femoral component of the TKR. Due to the severity of her presentation, empirical antibiotic therapy was commenced using intravenous vancomycin and doxycycline.

### Immediate surgical management

Over the following night, she continued to deteriorate into overt sepsis, with a qSOFA score of 2 due to the onset of hypotension and increasing confusion (AMTS 7/10) 3. On the morning of Day 11, she was taken to theatre to perform excision, debridement and washout of her thumb. During this operation, orthopaedic surgeons also performed sterile aspiration of the knee - revealing 25mL of frank pus. Due to haemodynamic instability, the patient was admitted to ICU for further optimisation before escalating towards further surgical management.

Once on ICU, the patient had a turbulent postoperative recovery. She experienced acute delirium (AMTS 3/10) and fluctuating consciousness, which was far from her baseline mental state. With continued deterioration, she was taken back to theatre for emergency arthroscopic joint washout on the evening of Day 11. She returned to ICU following the procedure, where she remained under critical care for a further 72 hours. Empirical antibiotics were subsequently changed to meropenem under the guidance of Lister microbiologists due worsening renal impairment.

### Post-operative Management

Over the next 72 hours, the patient's delirium and acute kidney injury improved, with comments of her appearing “systemically improved” first appearing in documentation four days post-operatively. Two blood cultures taken on admission returned with no detectable growth after enrichment. Furthermore, microbiology samples from her joint aspirate and arthroscopic washout both returned with no organisms detected.

Over the following week, she maintained improvements both clinically and biochemically. With visible prosthetic loosening on plain radiographs and the severity of her initial infection, it was felt that two-stage revision would be the most appropriate operative strategy. On day 22, she underwent first stage revision, with a temporary cement spacer and silver-based components inserted in place of her infected metalwork. Intraoperatively, the surgeon commented on extensive destructive bone loss, which had resulted in significant loosening of her cemented prosthesis.

After her revision procedure, the patient steadily improved day-by-day. She remained on carbapenem antibiotics as the mainstay of her antimicrobial management. On day 27, microbiology revealed that *Streptobacillus moniliformis* had been detected on operative tissue samples using 16S PCR.

### Discharge and Outpatient Management

For the remainder of her stay, the patient remained clinically asymptomatic with no concerning change in her blood results. Once therapy-fit, she was discharged home on Day 36 to complete a six-week course of intravenous carbapenem antibiotics under the hospital outpatient service.

She has received outpatient clinic reviews at four, six and eight weeks post-discharge. She has maintained a stable clinical and biochemical improvement at follow-up; with CRP remaining <10 post-discharge. Planning is now underway for her second-stage knee revision.

## Discussion

### Foundations of Streptobacillus moniliformis

*Streptobacillus moniliformis* is a highly pleomorphic filamentous gram-negative bacillus that was first isolated by Schotuller in 1914 4. It belongs to the *Leptotrichiaceae* family of bacteria, which has recently experienced an eruption of new discoveries. Since 2014, there have been four novel species discovered within the same family, termed *Streptobacillus hongkongensis*, *Streptobacillus felis*, *Streptococcus ratti* and *Streptobacillus notomytis* 5.

It is most commonly known to colonise the nasopharynx and bodily secretions of rodents, with 70-91% of infected individuals documenting contact with rodents 6,7. There are also reports of contagion through animals that prey on rats (e.g. cats), and from human-to-human oral transmission 8,9,10.

In previous literature, infection with *S. moniliformis* has been described as two related conditions. “Rat-bite fever” is generically defined as a febrile illness following a rodent bite. After suffering a rodent bite, there is a 10% risk of developing rat-bite fever - although it is unknown how many of these cases can be attributed specifically to *S. moniliformis* 11. Infection may follow assault not only from rats, but from other rodents such as ferrets, squirrels and guinea pigs 8. Secondly, systemic illness caused by *S. moniliformis* has been described under the eponym, Haverhill fever - although this is not necessarily contracted following a rodent bite.

Both rat-bite fever and Haverhill fever have been described as systemic presentations. There have been anecdotal cases of local septic arthritis and osteomyelitis attributed to *S. moniliformis*, but our case presents the first documented periprosthetic infection 6,7,12,13.

### Clinical Picture

As with most bacteraemic fevers, presentation is usually non-specific. As a result, clinical suspicion is raised through relevant social history and lifestyle factors. Greater risk is associated with frequent exposure to rodents; such as animal laboratory workers and those from a low socioeconomic backgrounds - particularly the homeless.

Polyarticular arthralgia is a major hallmark of *S. moniliformis* infection, present in 83% of cases. Articular reactions may become more pronounced, presenting as septic arthritis or autoimmune-mediated reactive arthritis. Most cases of septic arthritis are asymmetrical, with the knee being the most commonly affected joint in adults - and hip being more commonly affected in paediatric groups 6,13.

Alongside polyarthralgia, patients commonly complain of pyrexia (58%) and constitutional symptoms, such as vomiting, headaches and myalgia 11. 17% also experience a rash 6. Typically, these symptoms develop three days to three weeks following initial exposure 7.

Septic arthritis and osteomyelitis are known progressions of untreated bacteraemic infection. Further documented complications include malignant pneumonia, hepatitis, discitis, meningoencephalitis and endocarditis - the latter carrying a 43-53% mortality rate 7,8,11,14,15. Torres-Miranda highlighted the importance of identifying *S. moniliformis*, as most cases of fatal endocarditis received non-targeted antibiotic therapy. Overall, rat-bite fever carries a mortality rate of approximately 7-15% when left untreated 8,11.

### Detecting Streptobacillus moniliformis

Perhaps the most noteworthy characteristic of *S. moniliformis* is an ability to remain undetectable in commonly-used hospital blood cultures 12,16,17. Most blood culture mediums contain the anticoagulant, polyanethol sulfonate, which has been shown to inhibit the growth of *S. moniliformis* at concentrations greater than 0.02% 18,19. This predisposes to an exceedingly high false-negative rate.

In 2008, Kimura et al. compared detection of *S. moniliformis* in standard culture mediums versus 16S rRNA sequencing and polymerase chain reaction amplification, demonstrating a significant improvement in detection rate 20. As a result, molecular sequencing methods remain the gold standard for detection.

*S. moniliformis* bacteraemia is felt to be under-diagnosed primarily due to a lack of clinical awareness or suspicion amongst medical professsionals 11. The historical indications to perform PCR emphasises the importance of a clear and accurate clinical narrative. Any exposure to rodents or unidentified polyarticular reactive arthritis would be a clear trigger to indicate non-standard testing.

## Learning Points

While polyarticular reactions are well-documented, this novel case of rat-bite fever presented as a severe periprosthetic monoarthropathy with rapidly destructive progression. Although periprosthetic infection is an unfortunately common occurrence, there are no documented cases implicating *Streptobacillus moniliformis* 1.

In the case demonstrated, a delayed presentation and severity of sepsis indicated a more radical operative approach. While joint revision was undertaken on day 22, there was already noted to be macroscopically visible destruction of bony structures and loosening of prosthetic components. This suggests early intervention and low thresholds for escalation of operative strategies would be sensible in similar cases.

In addition, penicillin-based monotherapy has been the first line antimicrobial strategy in existing literature. There is no established second-line therapy, but anecdotal success has been documented using ampicillin or ceftriaxone monotherapy and nafcillin/ gentamicin or flucloxacillin/vancomycin combination regimes 6. In this case, we have evidenced carbapenems as a successful eradication regimen for patients with known type-1 penicillin hypersensitivity. Although there is no established antibiotic duration for *S. moniliformis* periprosthetic infection, a six-week course has proven sufficient for eradication in this case.

## Supplementary Material

Appendix 1: Timeline of events and antibiotic usage (days).

## Abbreviations

AKI: Acute kidney injury
AMTS: Abbreviated mental test score
CRP: C-reactive protein
CT: computer tomography
ICU: Intensive care unit
IV: intravenous
PCR: Polymerase chain reaction
PICC: Peripherally inserted central catheter
PIPJ: Proximal interphalangeal joint
PJI: Prosthetic joint infection
qSOFA: Quick sequential organ failure assessment
TKR: Total knee replacement
WCC: White cell count

## References

[1] Li C, Renz N, Trampuz A (2018). Management of periprosthetic joint infection. Hip & pelvis.

[2] Izakovicova P, Borens O, Trampuz A (2019). Periprosthetic joint infection: current concepts and outlook. EFORT open reviews.

[3] Marik PE, Taeb AM (2017). SIRS, qSOFA and new sepsis definition. Journal of thoracic disease.

[4] Schottmüller H (1914). Zur atiologie und klinik der bisskrankheit (ratten-, katzen-, eichhornchen-bisskrankheit). Dermatol Wochenschr.

[5] Ogawa Y, Kasahara K, Lee ST (2018). Rat-Bite Fever in Human with Streptobacillus notomytis Infection, Japan. Emerging infectious diseases.

[6] Wang TK, Wong SS (2007). Streptobacillus moniliformis septic arthritis: a clinical entity distinct from rat-bite fever?. BMC infectious diseases.

[7] Torres-Miranda D, Moshgriz M, Siegel M (2018). Streptobacillus moniliformis mitral valve endocarditis and septic arthritis: the challenges of diagnosing rat-bite fever endocarditis.

[8] Akter R, Boland P, Daley P (2016). Rat bite fever resembling rheumatoid arthritis.

[9] Place EH, Sutton LE (1934). Erythema arthriticum epidemicum (Haverhill fever). Archives of Internal Medicine.

[10] Fordham JN, McKay-Ferguson E, Davies A (1992). Rat bite fever without the bite. Annals of the rheumatic diseases.

[11] Gossman WG, Bhimji SS (2017). Rat-bite Fever (Streptobacillus moniliformis, Sodoku, Spirillum Minor).

[12] Wallet F, Savage C, Loïez C (2003). Molecular diagnosis of arthritis due to Streptobacillus moniliformis. Diagnostic microbiology and infectious disease.

[13] Wegner AM, Look N, Haus BM (2017). Surgical management of multijoint septic arthritis due to rat-bite fever in a pediatric patient: a case study.

[14] Washburn RG (2005). Streptobacillus moniliformis (rat-bite fever). Mandell GL, Bennett JE, Dolin R.

[15] Rupp ME (1992). Streptobacillus moniliformis endocarditis: case report and review. Clinical infectious diseases.

[16] Boot R, Oosterhuis A, Thuis HC (2002). PCR for the detection of Streptobacillus moniliformis. Laboratory animals.

[17] Berger C, Altwegg M, Meyer A (2001). Broad range polymerase chain reaction for diagnosis of rat-bite fever caused by Streptobacillus moniliformis. The Pediatric infectious disease journal.

[18] Graves MH, Janda JM (2001). Rat-bite fever (Streptobacillus moniliformis): a potential emerging disease. International journal of infectious diseases.

[19] Rygg M, Bruun CF (1992). Rat bite fever (Streptobacillus moniliformis) with septicemia in a child. Scandinavian journal of infectious diseases.

[20] Kimura M, Tanikawa T, Suzuki M (2008). Detection of Streptobacillus spp. in feral rats by specific polymerase chain reaction. Microbiology and immunology.

