# Supplementary figures and images for: Near-fatal Periprosthetic Infection with Streptobacillus moniliformis: Case and Review
Source: J Bone Jt Infect. 2020 Feb 21;5(1):50–3. doi: 10.7150/jbji.40635 (PMC7045529; doi:10.7150/jbji.40635)

## Appendices

### Appendix 1: Timeline of events and antibiotic usage (days)

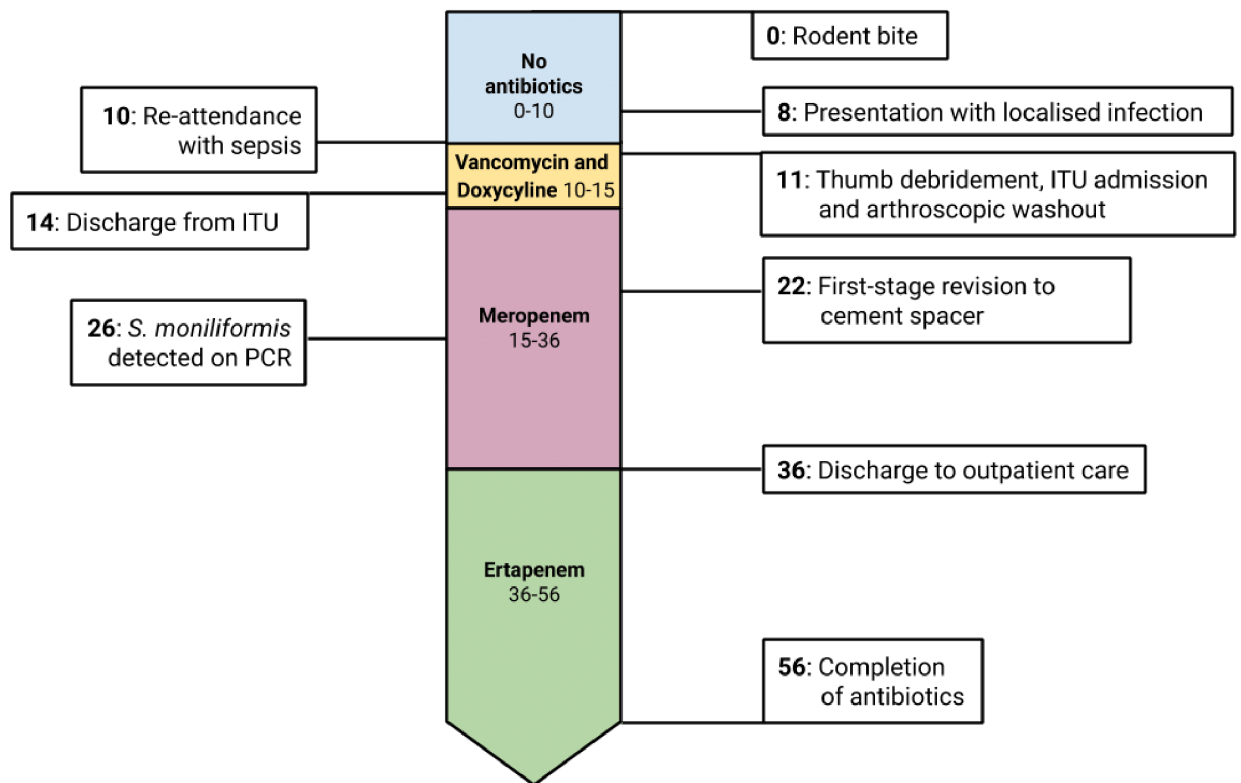

Supplement: Supplementary file 1 — Appendix 1: Timeline of events and antibiotic usage (days). [file jbjiv05p0050s1.pdf]
